# The Gti1/Pac2 family protein CFG1 controls fungal chlamydospore formation through orchestrating cell wall remodeling, lipid metabolism, and ribosome biogenesis

**DOI:** 10.1128/aem.00867-26

**Published:** 2026-08-05

**Authors:** Yiting Hou, Yifan Li, Cheng Chen, Xichen Ling, Minglei Cheng, Jianwei Pu, Tingting Sun, Jian Zhang, Qirong Shen, Zhenzhong Yu

**Affiliations:** 1 Jiangsu Provincial Key Lab for Solid Organic Waste Utilization, Jiangsu Collaborative Innovation Center for Solid Organic Wastes, Educational Ministry Engineering Center of Resource-Saving Fertilizers, Jiangsu Provincial Center for Agricultural Microbial Resource Protection and Germplasm Innovation and Utilization, Nanjing Agricultural University 70578, Nanjing, China; Chalmers tekniska hogskola AB, Gothenburg, Sweden

**Keywords:** lipid metabolism, dormancy, Gti1/Pac2 family, chlamydospores

## Abstract

The morphological transition of fungi from vegetative hyphae to thick-walled chlamydospores enhances their longevity in harsh environmental conditions. Owing to this resilience, pathogenic fungi that form chlamydospores are particularly difficult to control. Therefore, understanding the mechanisms of chlamydospore formation is critically important. Here, we show that the hyphae of the filamentous fungus *Trichoderma guizhouense* can differentiate into typical terminal and intercalary chlamydospores characterized by double-layered spherical or ellipsoidal cell walls with accumulated lipid bodies and nuclei. We found that during chlamydospore formation, ribosome biogenesis was gradually downregulated, indicating the entry of cells into dormancy. Comparative transcriptomic analyses across developmental stages and media identified the Gti1/Pac2 family protein CFG1 as an essential regulator, as the Δ*cfg1* strain failed to form chlamydospores under all inducing conditions. Lipidomic analysis showed its involvement in lipid metabolism, and mutants lacking lipid metabolism genes *pdat* or *dgat* produced fewer chlamydospores. Our work reveals the molecular mechanism of chlamydospore formation in *T. guizhouense*.

**IMPORTANCE:**

In fungal biology, the morphological transition from vegetative hyphae to thick-walled, lipid-rich chlamydospores represents a fundamental developmental switch into dormancy, crucial for survival under environmental stress. Understanding the regulatory mechanisms behind this process is essential for deciphering the basic principles of fungal cell differentiation and adaptation. This study employs multi-omics approaches to systematically characterize chlamydospore formation and identifies the Gti1/Pac2 family protein CFG1 as a master regulator. Functional analysis reveals that CFG1 governs this transition by directly influencing lipid metabolism—a key pathway for spore maturation and structural integrity. These findings uncover a previously unknown molecular switch in fungal development and provide new insights into how filamentous fungi coordinate metabolic reprogramming with cellular differentiation to ensure long-term survival.

## INTRODUCTION

Fungi utilize various strategies to survive in changing environments, one of which is the formation of dormant spores that can revive under favorable conditions (1). Spores are metabolically quiescent yet preparative during dormancy (2). In this state, spores exhibit extremely suppressed metabolism with low respiration and macromolecular synthesis rates while being enriched with energy-rich reserves, such as lipids, glycogen, and trehalose, to ensure long-term viability. These reserves are rapidly mobilized upon germination to fuel the initial growth. In contrast, dormant spores are protected by a thicker cell wall, which may contain additional layers or pigments that confer enhanced mechanical strength and chemical resistance (2). Chlamydospores are enlarged, thick-walled cells with condensed cytoplasm, typically formed within hyphae (intercalary chlamydospores) or at hyphal tips (terminal chlamydospores) (3). They exhibit high resistance to various stresses, such as low temperatures, unfavorable pH, desiccation, and nutrient deficiency (4). This robustness underpins fungal longevity (5, 6). Chlamydospores produced by soil-borne plant pathogenic fungi can survive for several years in the soil, making their control in agriculture more difficult (7, 8).

The formation of chlamydospores is normally initiated under extreme living conditions (9). Carbon starvation stimulates vegetative hyphae of the plant wilt-causing fungus *Fusarium oxysporum* to differentiate into dormant chlamydospores, which can survive in harsh soil environments. Once the exudates of banana roots are sensed, these chlamydospores germinate rapidly to infect the plant roots, giving rise to fusarium wilt of bananas (7). The human pathogenic yeast *Candida albicans* produces more chlamydospores when carbon sources are deficient, and these spores exhibit superior capabilities in skin surface colonization, adhesion, and drug resistance (10–12). The wheat pathogenic fungus *Zymoseptoria tritici*, which infects old leaves where nutrients have been exhausted, produces chlamydospores that are more resistant to desiccation and extreme temperatures than other types of asexual spores (4). Dehydration promotes chlamydospore formation in the nematode-trapping fungus *Duddingtonia flagrans* (13). In addition, some lipopeptides produced by bacteria and fungi induce chlamydospore formation. The lipopeptide ralsolamycin, synthesized by the soil-borne plant pathogenic bacterium *Ralstonia solanacearum*, can stimulate chlamydospore formation in more than 30 species of fungi (14). A few studies have investigated the molecular mechanisms of chlamydospore formation in the dimorphic human pathogenic yeasts *Cryptococcus neoformans* and *Candida albicans*. However, these mechanisms remain largely enigmatic in filamentous fungi that can form more typical terminal and intercalary chlamydospores (3).

The Gti1/Pac2 protein family is a class of fungal-specific transcriptional regulators, defined by a conserved N-terminal Gti1/Pac2 domain, that regulates the development and virulence of pathogenic fungi (15, 16). Wor1, the best-characterized member of the Gti1/Pac2 family, is a master regulator of the morphological transition between saprophytic and invasive growth in *C. albicans* (17–21). In *F. oxysporum*, the Gti1/Pac2 family protein Sge1 is essential for the expression of genes encoding effectors secreted during the infection process (22). In the rice blast fungus *Magnaporthe oryzae*, MoGti1 and MoPac2 of the Gti1/Pac2 family proteins demonstrate conserved functions in regulating vegetative growth, conidiation, effector gene expression, and pathogenicity (23). In the maize smut fungus *Ustilago maydis*, the Gti1/Pac2 family protein Ros1 is involved in teliospore formation and controls filamentation and pathogenicity (24).

*Trichoderma* spp. are filamentous fungi widely distributed in environments such as soil and plant residues (25, 26). They can promote plant growth and antagonize plant pathogenic fungi, and are thus widely used in agriculture (27). Owing to their resilience and longevity, chlamydospores are potentially ideal cell types for formulating effective *Trichoderma*-based plant growth stimulants and biocontrol agents (28). In this study, we found that the filamentous fungus *Trichoderma guizhouense* can form typical terminal and intercalary chlamydospores under certain nutrient conditions. We used *T. guizhouense* as a model organism to investigate the molecular mechanisms underlying chlamydospore formation in filamentous fungi. By comparative analysis of different transcriptomes, a key regulator, CFG1 (Chlamydospore Formation Gene), belonging to the Gti1/Pac2 protein family, was identified, and the underlying regulatory mechanism was further revealed.

## RESULTS

### *T. guizhouense* can form typical terminal and intercalary chlamydospores

**Fig 1:**
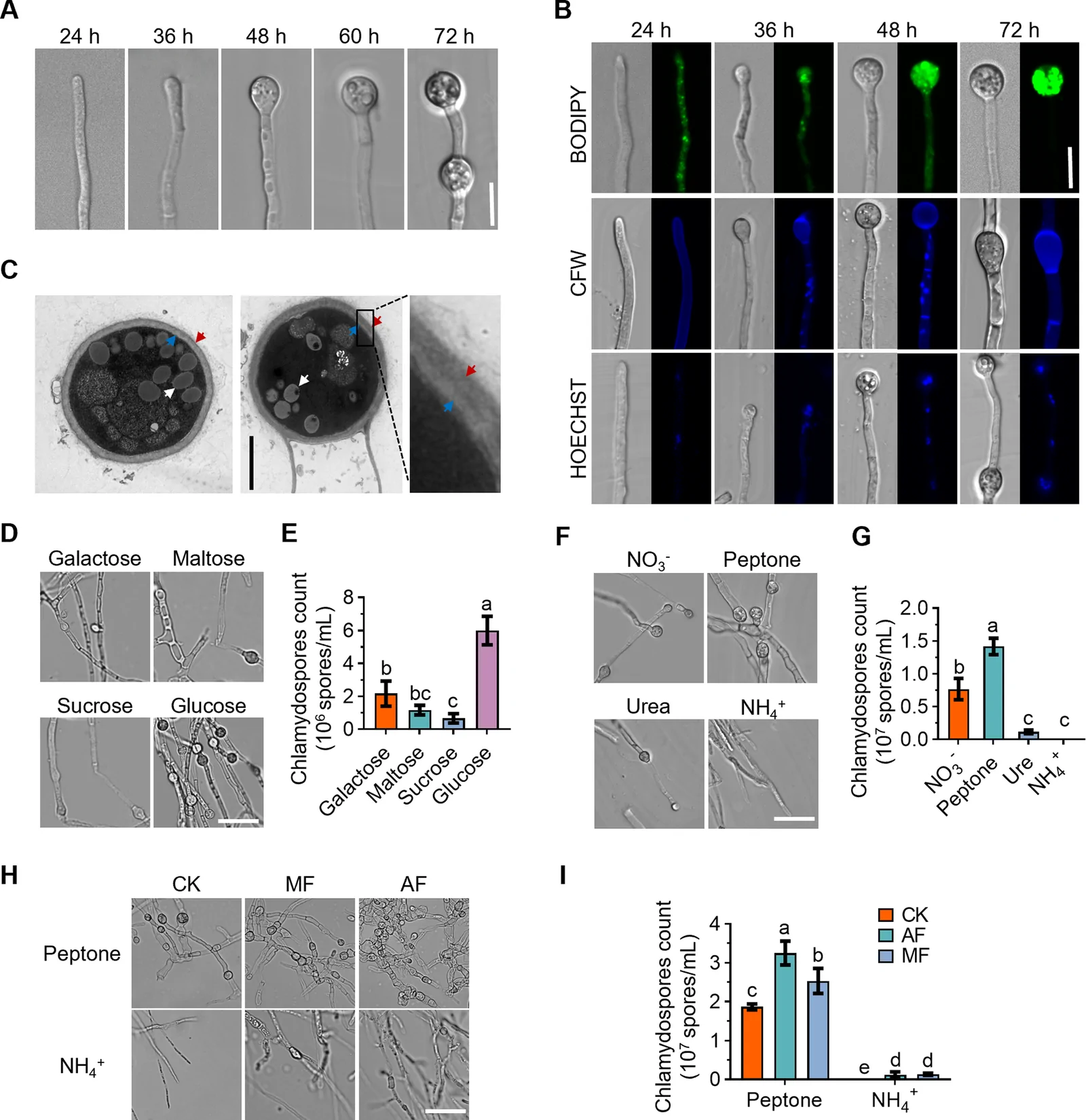
Morphological characterization and nutrient-dependent chlamydospore formation in *T. guizhouense*. (**A**) The process of mycelial change includes spore germination, hyphal growth, and asexual development. Scale bar, 5 μm. (**B**) Fluorescent staining of lipid droplets, cell walls, and nuclei. BODIPY solution was used for the detection of lipids. A CFW solution was used for cell wall detection. Hoechst solution was used for nuclear detection. Scale bar, 5 μm. (**C**) Transmission electron microscopy (TEM) image of chlamydospores. Red arrow, outer wall; blue arrow, inner wall; white arrow, lipid droplets. Scale bar, 2 μm. (**D**) Phenotype of wild-type (WT) strain in carbon sources. Wild type was grown at 28°C on a rotary shaker (170 rpm) for 120 h. Scale bar, 15 μm. (**E**) Quantification of chlamydospores produced by wild type on carbon sources. (**F**) Phenotype of the WT strain under nitrogen sources. Scale bar, 15 μm. (**G**) Quantification of chlamydospores produced by WT in nitrogen source medium. (**H**) Phenotype of the WT strain in peptone and ammonium sulfate medium supplemented with MF and AF, each at 0.4 μM. Scale bar, 15 μm. (**I**) Quantification of chlamydospores of wild type produced in peptone and ammonium sulfate medium supplemented with MF and AF. (**E, G, and I**) Error bars indicate the SD of three biological replicates. Statistical analysis was performed using one-way ANOVA followed by Tukey’s test, and different lowercase letters represent significant differences (*P* < 0.05). Groups not sharing any common letter are significantly different from each other.

Among the key strategies employed by fungi to withstand adverse conditions is the formation of chlamydospores from susceptible hyphae (29). We found that *T. guizhouense* produced typical terminal and intercalary chlamydospores when cultured in a liquid minimal medium at 28°C. We observed that the hyphal tips of the wild type started expanding after 36 h of cultivation, and mature terminal chlamydospores were formed after 60 h (Fig. 1A). Intercalary chlamydospores were formed after 72 h of cultivation. The formation of a dormant chlamydospore involves major restructuring of the cell wall to reshape a globose cell and comprehensive consolidation of resources, packing nuclei, and energy sources for dormancy and future germination (30). We stained the hyphae with BODIPY to analyze the dynamic changes in the lipid droplets and found that the fluorescent signals of lipid droplets gradually accumulated at the hyphal tips. Moreover, all lipid droplets accumulated in the globose mature chlamydospores after 72 h, and no signals were observed in the hyphae adjacent to the chlamydospores (Fig. 1B). To investigate how the cell wall was remodeled during this process, we stained the chitin with calcofluor white (CFW). Chitin deposition was detected in the chlamydospore cell wall at 48 h, and this deposition increased as the chlamydospores enlarged (Fig. 1B). The TEM images showed that the chlamydospores had a double-walled structure with a thin outer wall (red arrow) and a thick inner wall (blue arrow) (Fig. 1C). A large number of lipid droplets were also observed (white arrow). In addition, nuclei were observed inside the developing chlamydospores at 36 h but were absent from the adjacent hyphae (Fig. 1B). Taken together, *T. guizhouense* can form typical terminal and intercalary chlamydospores through a reconstructing process marked by the consolidation of lipid reserves and nuclei into a globose cell and remodeling of the cell wall into a thick, double-layered structure.

### Nitrogen source is more crucial than carbon source for chlamydospore formation

Nutritional conditions serve as critical environmental cues that affect chlamydospore formation (31). To analyze the effects of different nutrients on chlamydospore formation, the wild-type strain was cultured in modified minimal medium containing different carbon (galactose, maltose, sucrose, and glucose) or nitrogen (ammonium sulfate, peptone, nitrate, and urea) sources. After 5 days of cultivation at 28°C, the formation of chlamydospores under different nutritional conditions was compared. We found that glucose was the most preferred carbon source, and the amount of chlamydospores was significantly decreased when glucose was replaced with galactose, maltose, or sucrose (Fig. 1D and E). As for the nitrogen sources, peptone and nitrate promoted the formation of chlamydospores, whereas only a few spores were produced in the medium containing urea (Fig. 1F and G). Notably, no chlamydospores were formed when ammonium sulfate was the sole nitrogen source. These results indicate that the nitrogen source is crucial and that ammonium sulfate cannot induce chlamydospore formation. It has been reported that certain antifungal lipopeptides can induce chlamydospore formation in other fungi (32, 33). We found that the presence of micafungin (MF) and anidulafungin (AF) rescued chlamydospore formation in the otherwise non-permissive ammonium sulfate condition (Fig. 1H and I). Collectively, these results indicate that the nitrogen source is more critical than the carbon source for chlamydospore formation in *T. guizhouense*.

### Transcriptome reprogramming drives cellular differentiation during chlamydospore formation

**Fig 2:**
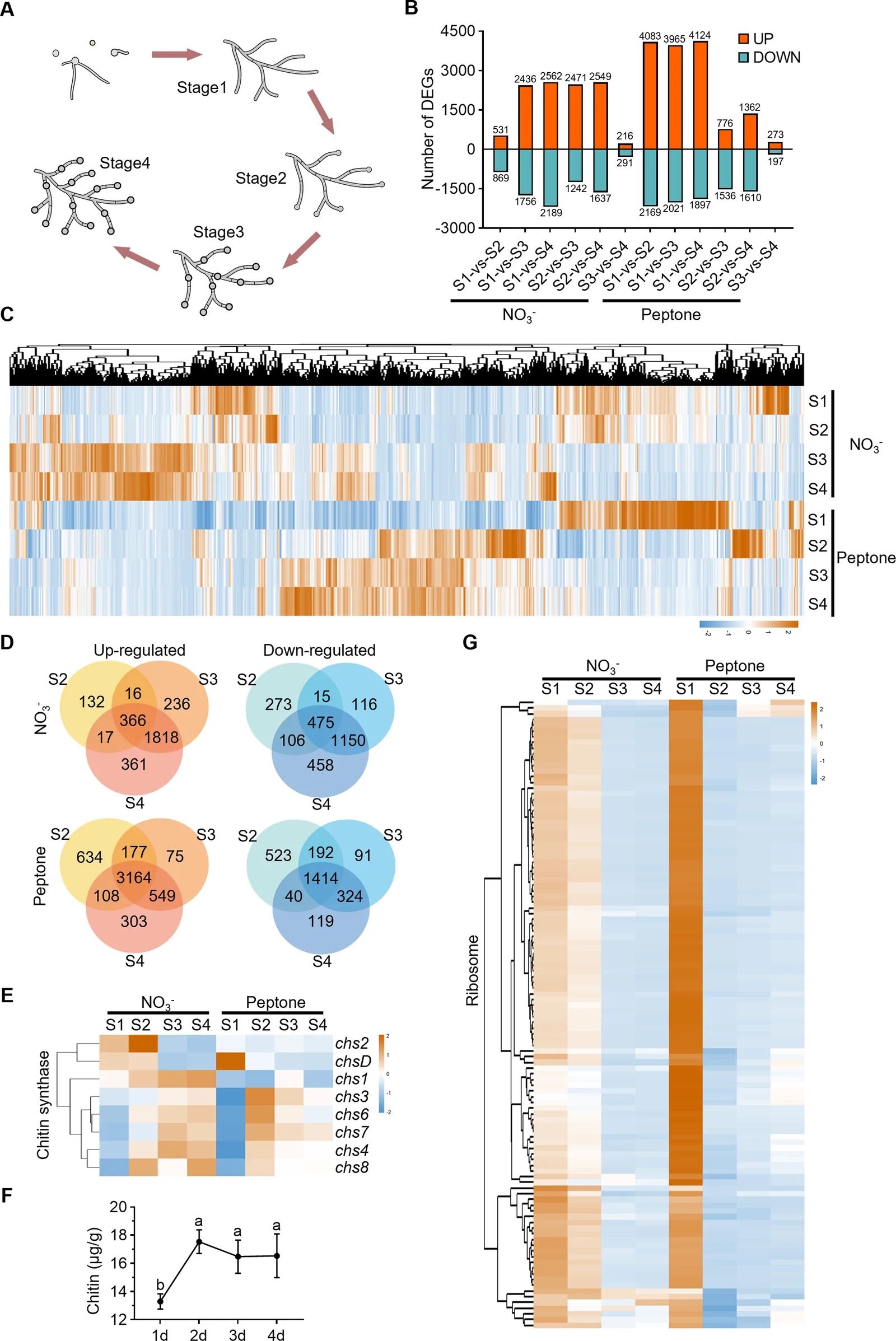
Transcriptional dynamics and key pathway regulation during chlamydospore development in *T. guizhouense*. (**A**) Life cycle of *T. guizhouense*, including spore germination, hyphal growth, and asexual development. (**B**) Distribution of upregulated and downregulated differentially expressed genes (DEGs) between adjacent time points in nitrate (NO_3_^−^) and peptone media. The number of DEGs is indicated above the histograms. (**C**) Genomic heatmap of DEGs relative to S1 under MM and peptone medium conditions. (**D**) Venn diagrams showing the overlap of upregulated and downregulated DEGs between developmental stages S2, S3, and S4 under NO_3_^−^ and peptone culture conditions. (**E**) Heatmap plots showing gene expression values and changes in chitin synthase genes in NO_3_^−^ and peptone media. (**F**) Mycelial chitin content in WT at 1–4 days. Error bars indicate the SD of three biological replicates. Statistical analysis was performed using one-way ANOVA followed by Tukey’s test, and different lowercase letters represent significant differences (*P* < 0.05). Groups not sharing any common letter are significantly different from each other. (**G**) Heatmap plots showing gene expression values and changes in ribosome genes in NO_3_^−^ and peptone media.

To investigate global gene expression changes during chlamydospore formation, transcriptome analysis was performed at four developmental stages (S1–S4) under two nitrogen sources (nitrate and peptone) (Fig. 2A). Surprisingly, compared with stage S1, ~42.4% (5,444/12,829) of the genomic genes in sodium nitrate medium and ~59.2% (7,623/12,829) in peptone medium were differentially expressed, indicating a genome-wide gene expression reprogramming during the formation of chlamydospores (Fig. 2B). In the sodium nitrate medium, 1,400, 4,192, and 4,751 DEGs were identified in stages S2, S3, and S4, respectively, compared to 6,252, 5,986, and 6,021 DEGs in the peptone medium. Among these, the expression levels of a substantial number of genes were consistently altered, whereas new sets of DEGs emerged at each stage (Fig. 2C and D). This pattern suggests continuous and dynamic reprogramming of genomic gene expression during chlamydospore formation. Next, GO enrichment analysis revealed a progressive shift from membrane-associated processes to metabolic activity and finally to cell wall remodeling during chlamydospore development (Fig. S1). Consistently, chitin synthase genes were upregulated at S3 and S4 (Fig. 2E), accompanied by increased chitin content (Fig. 2F). In nitrate medium, KEGG analysis of 841 co-regulated DEGs (366 up/475 down) revealed significant enrichment of pathways related to amino acid metabolism, fatty acid biosynthesis, and glycerophospholipid metabolism (Fig. S2A). In peptone medium, analysis of 4,578 DEGs (3,164 up/1,414 down) showed significant enrichment of ribosome, oxidative phosphorylation, protein processing, and glycerolipid metabolism pathways (Fig. S2B). Furthermore, in nitrate medium, 2,184 co-upregulated and 1,625 co-downregulated genes were identified in stages S3/S4, compared to 382 co-upregulated and 490 co-downregulated genes in stages S2/S3 (Fig. 2D). KEGG analysis of the 2,184 co-upregulated genes further confirmed enhanced activation of core metabolic processes, including amino acid metabolism, glycolysis/gluconeogenesis, fatty acid degradation, and glycerolipid metabolism (Fig. S2C). Taken together, these findings implicate energy and lipid metabolism in the process of chlamydospore formation.

Chlamydospores are vital asexual reproductive resting cells with low metabolic activity and gene expression (34). Ribosomes are sequestered in a translationally inactive state in dormant cells (35). Moreover, dormant cells can store mRNAs and ribosomes to quickly respond to recovery signals (36). Consistent with this, we observed significant enrichment of ribosome and oxidative phosphorylation pathways among downregulated genes (Fig. S2B and D), along with a progressive decline in ribosomal gene expression from early to late stages (Fig. 2G). These results confirm that chlamydospore formation is accompanied by a marked downregulation of energy metabolism and protein synthesis, reflecting a transition toward dormancy.

### The Gti1/Pac2 family transcription factor CFG1 is essential for chlamydospore formation

**Fig 3:**
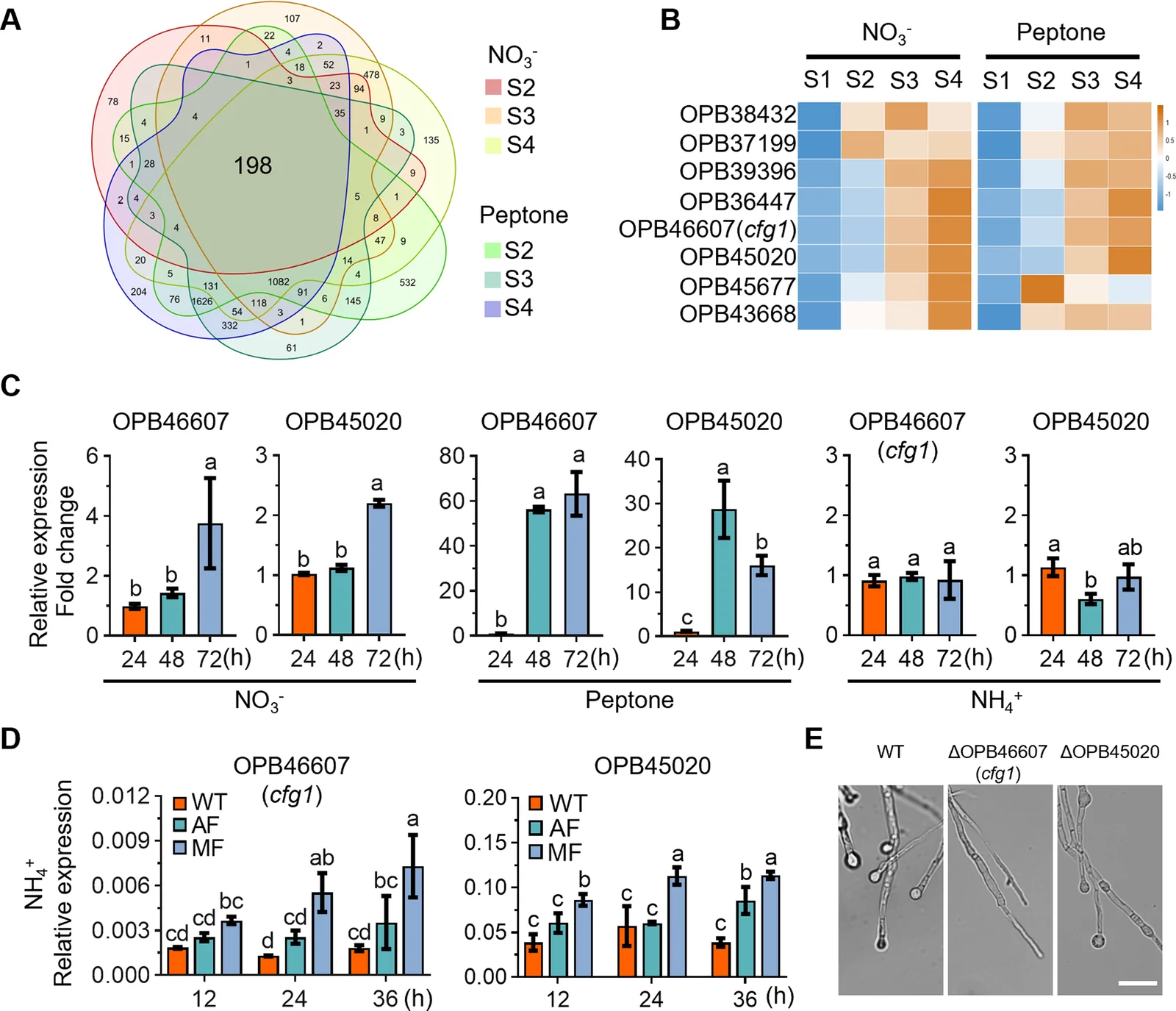
Identification and functional characterization of key transcription factors regulating chlamydospore formation. (**A**) Venn diagram of upregulated DEGs in S2, S3, and S4 cultured in NO_3_^−^ and peptone media. (**B**) Transcriptional profiles of OPB38432, OPB37199, OPB39396, OPB36447, OPB46607, OPB45020, OPB45677, and OPB43668 from transcriptome data in NO_3_^−^ and peptone media. (**C**) Expression levels of OPB46607 and OPB45020 in NO_3_^−^, peptone, and ammonium sulfate media after 3 days. (**D**) Expression levels of OPB46607 and OPB45020 in ammonium sulfate medium supplemented with MF and AF. (**E**) Phenotype of the WT, ΔOPB46607-, and ΔOPB45020-mutant, in NO_3_^−^ medium after 5 days. Scale bar, 15 μm. (**C and D**) Error bars indicate the SD of three biological replicates. Statistical analysis was performed using one-way ANOVA followed by Tukey’s test, and different lowercase letters represent significant differences (*P* < 0.05). Groups not sharing any common letter are significantly different from each other.

Next, we hypothesized that there might be some key regulators essential for chlamydospore formation. To identify these regulators, Venn diagram analysis of the upregulated DEGs from different stages in both media was performed. We found that a core set of 198 genes was co-regulated across all developmental stages under both culture conditions (Fig. 3A), and notably, only eight of them encode putative transcription factors (Fig. 3B). Since the strain did not produce chlamydospores in the minimal medium using ammonium sulfate as the sole nitrogen source, the expression level of the putative key transcription factor-encoding gene should not be upregulated under this culture condition. Indeed, reverse transcription quantitative PCR (RT-qPCR) results showed that two genes, OPB46607 and OPB45020, were no longer upregulated in the ammonium sulfate medium (Fig. 3C; Fig. S3). Moreover, the two genes were induced by the antifungal drugs micafungin (MF) and anidulafungin (AF) (Fig. 3D). To further analyze whether they are involved in chlamydospore formation, we deleted the two genes in the wild type separately and found that the mutant lacking OPB46607 could not produce chlamydospores, whereas the lack of OPB45020 did not affect chlamydospore formation (Fig. 3E). This result indicates the role of OPB46607 in chlamydospore formation. Therefore, the OPB46607 gene was named CFG1 (chlamydospore formation gene 1).

**Fig 4:**
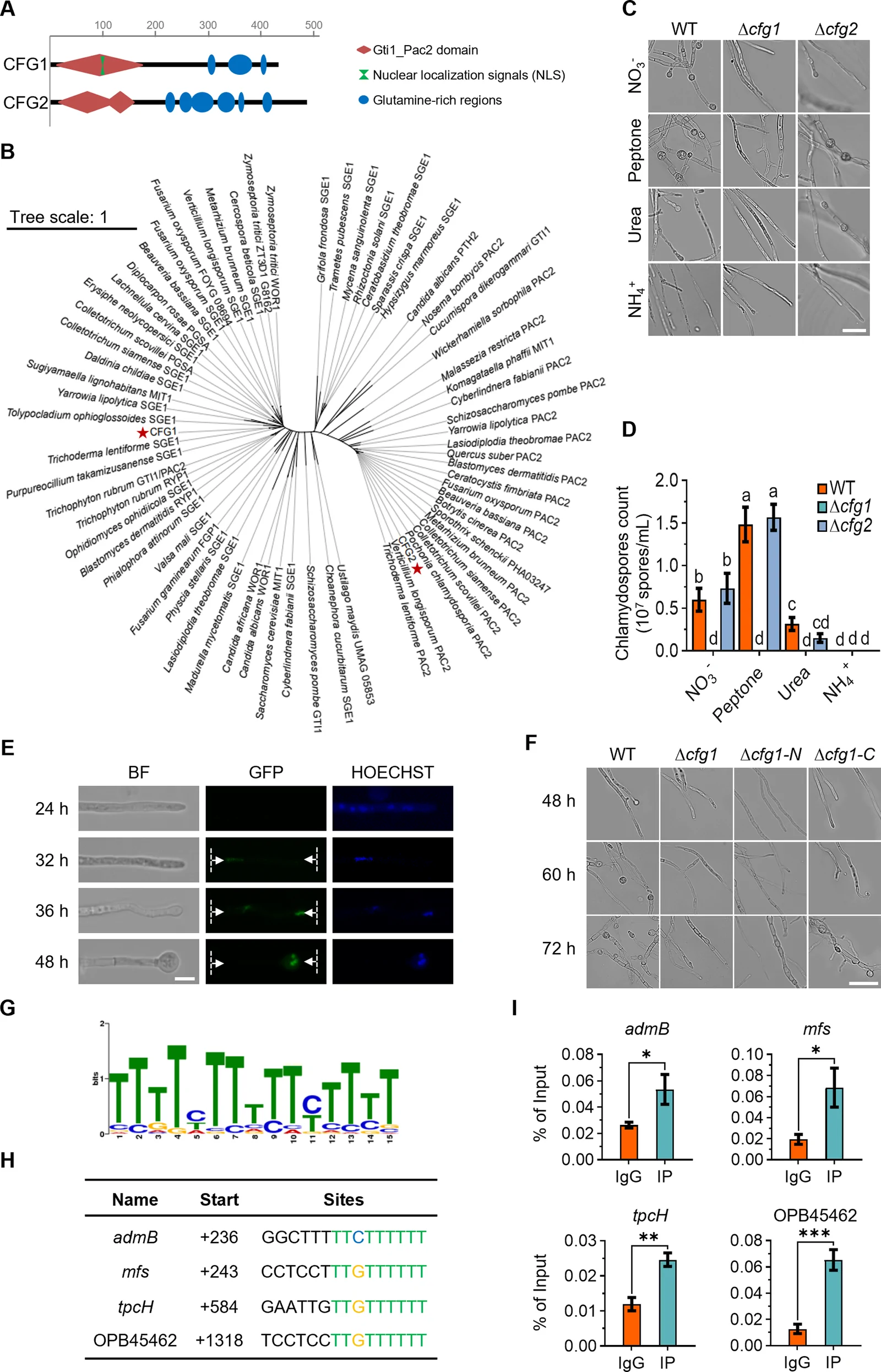
Roles and molecular mechanisms of Gti1/Pac2 family transcription factor CFG1 in chlamydospore development. (**A**) Functional analysis of Gti1/Pac2 family domains in *Trichoderma*. (**B**) Phylogenetic tree of CFG1 and CFG2 homologs from fungi. (**C**) Phenotypes of WT, Δ*cfg1*-, and Δ*cfg2*-mutant, on peptone medium after 5 days. Scale bar, 15 μm. (**D**) Quantification of chlamydospores under the above conditions. Error bars indicate the SD of three biological replicates. Statistical analysis was performed using one-way ANOVA followed by Tukey’s test, and different lowercase letters represent significant differences (*P* < 0.05). Groups not sharing any common letter are significantly different from each other. (**E**) Cellular localization of CFG1 at different times. EGFP fusion of the CFG1 protein was analyzed using fluorescence microscopy. Hoechst solution was used for nucleus detection. Scale bar, 5 μm. (**F**) Phenotypes of the Δ*cfg1* N-terminus and C-terminus mutants in peptone medium. Scale bar, 20 μm. (**G**) Motif recognized by CFG1. The motif and logo were developed using MEME. (**H**) Summary of the four selected candidate target genes and the location of their CFG1-binding motifs. (**I**) ChIP-qPCR for conserved motifs in *abmB*, *mfs*, *tpcH*, and OPB45462. Error bars indicate the SD of three biological replicates. Significant differences were evaluated using Student’s *t*-test: *P < 0.05; **P < 0.01; and ***P < 0.001.

The *cfg1* gene contains a 1,272-nucleotide open reading frame without introns, encoding a 423-amino acid protein. This protein consists of a canonical Gti1/Pac2 family domain (residues 10–171), a nuclear localization signal (PPGEKKR, residues 93–99) in its N-terminal region, and an unusually long polyglutamine stretch in its C-terminal region (Fig. 4A). Notably, the *T. guizhouense* genome encodes two putative Gti1/Pac2 family proteins. The second Gti1/Pac2 family protein, CFG2 (OPB36766), is characterized by two overlapping Gti1/Pac2 domains (residues 14–124 and 102–157) and shares only 35.48% sequence identity with the conserved domain of CFG1 (Fig. 4A). Further phylogenetic analysis of 68 Gti1/Pac2 family members from 51 fungi revealed that CFG1 clustered in the same clade as Sge1, whereas CFG2 was attributed to the Pac2 group (Fig. 4B). To determine whether both genes are involved in chlamydospore formation, we separately knocked out *cfg1* and *cfg2*, yielding Δ*cfg1* and Δ*cfg2* mutant strains (Fig. S4A). Microscopic examination revealed that the Δ*cfg1* mutants did not produce any chlamydospores after 5 days of culture on any of the media tested (Fig. 4C and D). In contrast, the Δ*cfg2* mutant still produced chlamydospores in liquid media with peptone, nitrate, or urea as the sole nitrogen source, although chlamydospore formation was delayed (Fig. 4C and D; Fig. S4D). Moreover, the addition of antifungal drugs (AF and MF) to the medium failed to rescue the chlamydospore-deficient phenotype of the Δ*cfg1* mutants (Fig. S4C). Additionally, we complemented the Δ*cfg1* mutant with the *cfg1* gene and constructed *cfg1* overexpression strains. Chlamydospore formation was restored in the complemented strain Δ*cfg1^C^*, and more chlamydospores were produced in the overexpression strain Δ*cfg1^OE^* than in the wild type (WT). These results indicate the decisive role of CFG1 in chlamydospore formation.

### CFG1 expression is progressively upregulated to directly regulate gene expression in the nucleus during chlamydospore formation

To analyze the localization of CFG1, a GFP strain expressing a C-terminal eGFP fusion of CFG1 was generated. After 24 h of cultivation, no GFP fluorescence was detected. After 32 h, weak GFP signals were observed in the nuclear region. After 36 h, hyphal tip swelling was evident, accompanied by the accumulation of GFP signal in the nuclei at the tip. Upon maturation of the terminal chlamydospore at 48 h, GFP fluorescence intensity markedly increased, reaching its maximum level, as confirmed by fluorescence intensity profiling (Fig. 4E and Fig. S4E). These observations indicate that CFG1 expression is progressively upregulated during chlamydospore formation. The N-terminal Gti1/Pac2 domain of CFG1 is fungal-specific and involved in the transcriptional control of morphogenesis (17, 23, 37–39), and the C-terminal region is intrinsically enriched in glutamine residues, forming a long polyglutamine (polyQ) stretch. However, the function of the C-terminal region remains unclear. To analyze the function of the C-terminal region, we constructed N-terminal and C-terminal deletion strains separately (Fig. S4B). The phenotype of the N-terminal deletion strain was consistent with that of Δ*cfg1* mutants. In contrast, chlamydospore production was delayed in the C-terminal deletion mutants (Fig. 4F). These results demonstrate that the C-terminal polyQ stretch also plays a regulatory role in chlamydospore formation.

In the dimorphic human pathogens *C. albicans* and *Histoplasma capsulatum*, Gti1/Pac2 family transcription factors specifically bind to the conserved promoter motif 5′-TTAKKSTTT-3′ (40). To test whether CFG1 binds to a similar motif, we employed DNA affinity purification sequencing (DAP-seq), a transcription factor (TF)-binding site (TFBS) discovery assay that couples affinity-purified TFs with next-generation sequencing of a genomic DNA library (41). Motif enrichment analysis of the sequencing data revealed several significantly enriched DNA sequence motifs (Fig. S5A). The CFG1 DNA-binding motif with the lowest *E* value (1.1E−113) was a 15 bp “TTNTHTTNTTBTTTT” binding region (Fig. 4G), in which the TTNTTBTTT motif is highly similar to that identified in *C. albicans* and *H. capsulatum*. To further validate the binding of CFG1 to this motif, we performed chromatin immunoprecipitation (ChIP) assays using a CFG1-eGFP strain, and primers were designed to amplify the promoter regions containing the TTNTTBTTT motif in four candidate target genes: *admB* (zinc-dependent metalloprotease), *mfs* (MFS permease), *tpcH* (glutathione S transferase), and OPB45462 (hypothetical protein) (Fig. 4H). Quantitative PCR (qPCR) analysis demonstrated significant enrichment of these promoter fragments in the ChIP samples from the CFG1-eGFP strain compared to the negative control for non-specific antibody interactions (Fig. 4I). Furthermore, no significant enrichment was observed for the control promoters (*atnl*, sphingoid long-chain base transporter; *bud2*, inhibitory regulator protein; and *fcr1*, Zn₂Cys₆ transcriptional regulator) lacking the CFG1-binding motif (Fig. S5B). Therefore, CFG1 serves as a transcription factor in the nucleus that directly regulates gene expression.

### CFG1 regulates lipid metabolism and storage during chlamydospore formation

**Fig 5:**
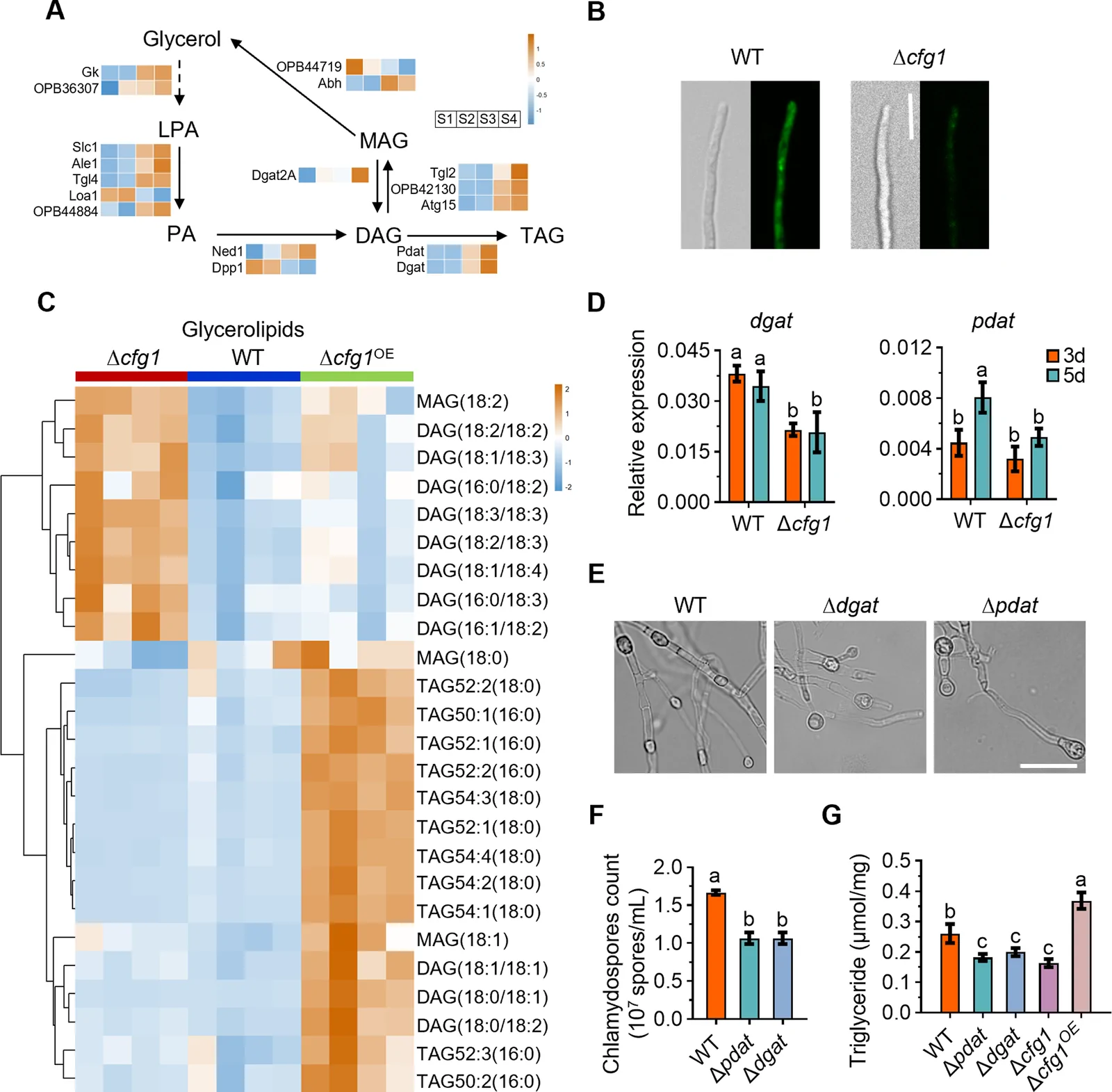
CFG1-mediated lipid metabolism is important for chlamydospore morphogenesis. (**A**) Transcriptome analysis of glycerolipid metabolism in response to NO_3_^−^ medium. The fragments per kilobase of transcript per million mapped reads (FPKM) values of glycerolipid metabolism genes were used to create a heatmap using the Heatmap package in RStudio. (**B**) Fluorescent staining of lipid droplets. BODIPY solution was used for lipid detection in the WT and Δ*cfg1* mutants. Scale bar, 5 μm. (**C**) Heatmap analyses of GLs species in WT, Δ*cfg1*, and Δ*cfg1^OE^*. (**D**) Expression levels of *dgat* and *pdat* in peptone medium in WT and Δ*cfg1* after 5 days. (**E**) Phenotypes of the WT, Δ*dgat*, and Δ*pdat* strains in peptone medium. Scale bar, 15 μm. (**F**) Quantification of chlamydospores of WT, Δ*dgat*, and Δ*pdat* strains produced in peptone medium after 5 days. (**G**) Intracellular triglyceride levels in WT, Δ*dgat*, Δ*pdat*, Δ*cfg1*, and Δ*cfg1^OE^* at 5 days. (**D, F, and G**) Error bars indicate the SD of three biological replicates. Statistical analysis was performed using one-way ANOVA followed by Tukey’s test, and different lowercase letters represent significant differences (*P* < 0.05). Groups not sharing any common letter are significantly different from each other.

Before entering the dormant state, chlamydospores must store sufficient energy reserves to sustain long-term survival under unfavorable conditions. Consistently, lipid droplets, the primary form of energy storage, accumulated in the chlamydospores (Fig. 1B), and transcriptomic analysis also revealed a comprehensive upregulation of genes involved in glycerolipid metabolism during S3 and S4 in the nitrate medium (Fig. 5A). Specifically, *gk* and OPB36307, which are responsible for converting glycerol into lysophosphatidic acid (LPA), were upregulated, suggesting an enhanced substrate supply for lipid synthesis. Enzymes catalyzing subsequent acylation reactions, including *slc1*, *ale1*, *tgl4*, and OPB44884, which convert LPA to phosphatidic acid (PA), also exhibited strong induction. Similarly, genes involved in the conversion of PA to diacylglycerol (DAG) (*ned1*) and DAG to triacylglycerol (TAG) formation (*pdat* and *dgat*) exhibited marked upregulation, highlighting an intensified TAG biosynthetic flux. This enhanced glycerolipid metabolism during S3 and S4 ensures that chlamydospores accumulate sufficient triacylglycerol reserves to support long-term survival and dormancy under nutrient-limited or stressful conditions. We further hypothesized that CFG1 plays an important role in lipid metabolism. To test this, we compared the lipid droplet accumulation in the WT and Δ*cfg1* strains (Fig. 5B). Compared with the WT, the fluorescence signal in the Δ*cfg1* mutant was significantly reduced, indicating a drastic decrease in lipid droplet levels. This suggests that CFG1 is critical for lipid storage and may play a key role in energy homeostasis during development.

Next, we performed lipidomic analysis on the strains, wild type, Δ*cfg1*, and Δ*cfg1^OE^*, that were cultured for 48 h in peptone medium. Based on the PLS-DA model, further refinement was performed using orthogonal partial least squares discriminant analysis (OPLS-DA) to obtain the OPLS-DA model plot and variable importance in projection (VIP) values. Lipid metabolites with a VIP value >1 for the first principal component of the OPLS-DA model in each comparison group, along with a univariate analysis *t*-test *P* < 0.05, were identified as significantly different lipid metabolites. Further analyses of the GLs showed that the Δ*cfg1* mutant exhibited a marked accumulation of diacylglycerols (DAGs) (Fig. 5C). This suggests a metabolic disruption, wherein the loss of *cfg1* impedes the efficient conversion of DAGs to triacylglycerols (TAGs). Conversely, the Δ*cfg1^OE^* strain exhibited an even greater abundance of TAGs than the WT (Fig. 5C), most likely due to increased activity or transcription of enzymes that catalyze the final step of TAG synthesis. These data indicate that CFG1 plays a critical role in regulating the flux of glycerolipid metabolism, promoting the synthesis and accumulation of neutral storage lipids (TAGs) from their precursor DAGs.

### Lipid metabolism genes regulated by CFG1 are involved in chlamydospore formation

In glycerolipid synthesis, TAG is formed through a reaction in which acyl-CoA serves as the acyl donor and DAG acts as the acceptor (42). This process is catalyzed by the enzyme phospholipid:diacylglycerol acyltransferase (PDAT). Diacylglycerol O-acyltransferase (DGAT) is a member of the membrane-bound O-acyltransferase (MBOAT) enzyme family that catalyzes the final step of triglyceride biosynthesis using acyl-CoA as a substrate (43). RT-qPCR results showed that the expression levels of these genes at 3 or 5 days were decreased in the Δ*cfg1* strain compared to those in the wild type (Fig. 5D). We constructed deletion strains Δ*pdat* and Δ*dgat* and quantified the chlamydospores. Although they still produced chlamydospores, the amounts were significantly reduced compared with those of the WT (Fig. 5E and F). We further examined the triglyceride concentrations in the mycelia of the wild type, Δ*pdat*, Δ*dgat,* Δ*cfg1*, and Δ*cfg1^OE^* strains. Compared with the WT, the concentration of triglycerides in the mycelia of all deletion strains decreased significantly (Fig. 5G). The triglyceride concentration in the Δ*cfg1^OE^* strain was significantly increased. These results indicate that both lipid metabolism genes play important roles in the formation of chlamydospores.

## DISCUSSION

Chlamydospores are of profound importance in the life cycle of many filamentous fungi, enabling them to withstand prolonged periods of environmental stress, such as nutrient deprivation, drought, extreme temperatures, and exposure to fungicides (3). This resilience allows pathogenic fungi to persist in soil or plant debris for years, serving as a primary inoculum source for new infection cycles and making diseases notoriously difficult to control (7). Therefore, understanding the mechanisms of chlamydospore formation is crucial for developing strategies to inhibit the formation or disrupt the dormancy of these resilient pathogenic fungi. However, these mechanisms in filamentous fungi remain largely underexplored. Here, we show that *T. guizhouense* produces typical terminal and intercalary chlamydospores, making it a good research model. Chlamydospore formation involves orchestrated morphological and physiological changes, including hyphal swelling, nuclear accumulation or clustering, lipid droplet deposition, cell wall thickening, and spore dormancy (28). Indeed, *T. guizhouense* forms spherical or ellipsoidal terminal chlamydospores first and then intercalary chlamydospores, during which lipid bodies and nuclei accumulate and ribosome biogenesis is gradually downregulated.

Nutrient cues, including nitrogen source type, carbon availability, and general nutrient limitation, are well-recognized triggers of sporulation, resting-cell formation, and morphological transitions in fungi (29). In *F. oxysporum*, chlamydospore production decreases with increasing NH_4_^+^ concentrations in the medium (44). We found that nitrogen sources are more crucial than carbon sources for chlamydospore formation. Peptone and nitrate facilitate their formation, whereas ammonium sulfate cannot induce this process. Notably, bacterial lipopeptides, such as ralsolamycin, can induce chlamydospore formation in many fungal species, such as Ascomycota, Basidiomycota, and Zygomycota (14). Consistent with this, lipopeptides can stimulate chlamydospore production in *T. guizhouense*, suggesting a conserved mechanism across fungi.

Extensive transcriptional repatterning is an established hallmark of fungal cellular differentiation, as observed during conidiation in *Aspergillus nidulans* and appressorium formation in *M. oryzae* (45, 46). Likewise, chlamydospore formation is driven by profound genome-wide transcriptional reprogramming, involving the differential expression of approximately 42.4% and 59.2% of all detected genes under nitrate and peptone conditions, respectively. This genome-wide transcriptional reprogramming directly facilitates key morphological changes, most notably in cell wall reinforcement. The upregulation of six chitin synthase genes during Stages S3 and S4 led to a significant increase in chitin content, forming another layer of the cell wall. Thickened cell walls confer mechanical strength and environmental resilience (47). Transcriptional reprogramming not only directs the structural fortification of the cell but also initiates a metabolic shutdown to enable dormancy. The downregulation of the ribosome and oxidative phosphorylation pathways observed here mirrors the hallmark of dormancy: suppression of global protein synthesis and ATP production to conserve energy (35, 36). Ribosome gene expression decreased gradually from stages S1 to S4 under nitrate and sharply after stage S1 under peptone, confirming a metabolic shift toward a dormant state. These findings are in line with the concept of hibernating ribosomes, which are translationally inactive but preserved for rapid reactivation under favorable conditions (36).

The Gti1/Pac2 family is known to control diverse biological processes including morphogenesis, cell cycle, stress response, and pathogenicity (40, 48–54). In *U. maydis*, loss of the Gti1/Pac2 protein Ros1 disrupts the formation of dark pigmented teliospores (24), while in *C. albicans*, the Gti1/Pac2 protein Wor1 controls white-opaque switching through a positive feedback mechanism that regulates its own expression (55–57). Although these studies focused on different morphological structures, they collectively suggest that Gti1/Pac2 family proteins govern the formation of dormant or stress-resistant cell types across diverse fungal species. Consistently, deletion of *cfg1* completely abolished chlamydospore formation, which could not be restored by the addition of micafungin and anidulafungin, highlighting its essential role in this process. Taken together, these findings emphasize the evolutionarily conserved role of the Gti1/Pac2 family proteins in regulating fungal morphological transitions. Whether these proteins are involved in regulating chlamydospore formation in other fungi warrants further investigation. Furthermore, we found that deletion of the N-terminal Gti1/Pac2 domain resulted in a phenotype identical to that of the Δ*cfg1* mutant, confirming that this domain is indispensable for CFG1 function. In contrast, deletion of the C-terminal polyQ stretch of CFG1 led to a delay in chlamydospore formation, suggesting a regulatory role. Similarly, in *Cladosporium fulvum*, the functional divergence of Wor1-like proteins, governed by their C-terminal regions, is critical for spore production and morphology (58). In *F. oxysporum* and *F. graminearum*, the regulation of pathogenicity by structurally conserved Wor1-like proteins is likely driven by their C-terminal regions (59).

We identified CFG1 as a nuclear-localized transcription factor, the DNA-binding motif (TTNTTBTTT) of which is highly similar to the “TTAKKSTTT” motif for Gti1/Pac2 family proteins in *C. albicans* and *H. capsulatum* (40). It directly governs the expression of three genes encoding a zinc-dependent metalloprotease (*admB*), an MFS permease (*mfs*), and a glutathione S-transferase (*tpcH*), which are associated with membrane transport, secondary metabolism, and antioxidant defense, respectively (60, 61). Glutathione S-transferase (GST) may contribute to the stress resistance of chlamydospores. As evidenced in *Lasiodiplodia theobromae*, specific GSTs (such as LtGST4 and LtGST9) can confer resistance to multiple fungicides by directly metabolizing and neutralizing these toxic compounds. GSTs also play a crucial role in mitigating oxidative stress through their peroxidase activity, reducing harmful peroxides that accumulate under adverse conditions (62).

TAGs are the principal form of neutral lipid storage in arbuscular mycorrhizal fungi (63). These lipids accumulate notably in spores, where they reach concentrations markedly higher than those in hyphae, serving as a crucial energy reserve to fuel spore germination (64). Although CFG1 does not directly regulate lipid metabolism-related genes, it profoundly influences lipid metabolic pathways. In Δ*cfg1*, the abundance of DAG was markedly elevated, whereas that of TAG was significantly reduced, indicating an obstruction in the biosynthetic pathway from DAG to TAG. This result is consistent with the observation that the Δ*cfg1* mutant did not form abundant lipid droplets. Previous studies have shown that PDAT overexpression in *S. cerevisiae* significantly increases TAG content (42). Similarly, in *Mortierella alpina*, MaDGAT1A and MaDGAT1B effectively promote TAG accumulation (65). Consistently, in our study, targeted deletion of the two downstream glycerolipid metabolism genes, *pdat* and *dgat*, resulted in a marked reduction in chlamydospore formation and a significant decrease in TAG levels. Taken together, these results reinforce the notion that the efficient conversion of DAG to TAG is crucial for lipid droplet formation.

An intriguing question for future investigation is how *cfg1* expression is activated by nutrient conditions to initiate chlamydospore formation. Given that chlamydospore formation typically occurs under nutrient-limited environments, it is plausible that *cfg1* is regulated by conserved nutrient-sensing cascades, such as the cAMP-PKA or TOR signaling pathways. Deciphering the upstream regulators of *cfg1* will provide critical insights into the environmental control of chlamydospore development. On the other hand, although the present study establishes CFG1 as an essential regulator of chlamydospore morphogenesis, whether CFG1-mediated chlamydospore formation directly contributes to the enhanced stress tolerance of *T. guizhouense* remains to be experimentally validated. Future studies employing stress tolerance assays (e.g., heat, UV, or oxidative stress) comparing the wild-type and Δ*cfg1* strains will be necessary to directly link CFG1 function to environmental fitness. Furthermore, the potential roles of CFG1-regulated lipid metabolism genes (such as *pdat* and *dgat*) and downstream target genes (including *tpcH*, which encodes a glutathione S-transferase) in stress resistance also warrant detailed investigation, as these pathways may contribute to the resilience of chlamydospores through both energy storage and detoxification mechanisms.

**Fig 6:**
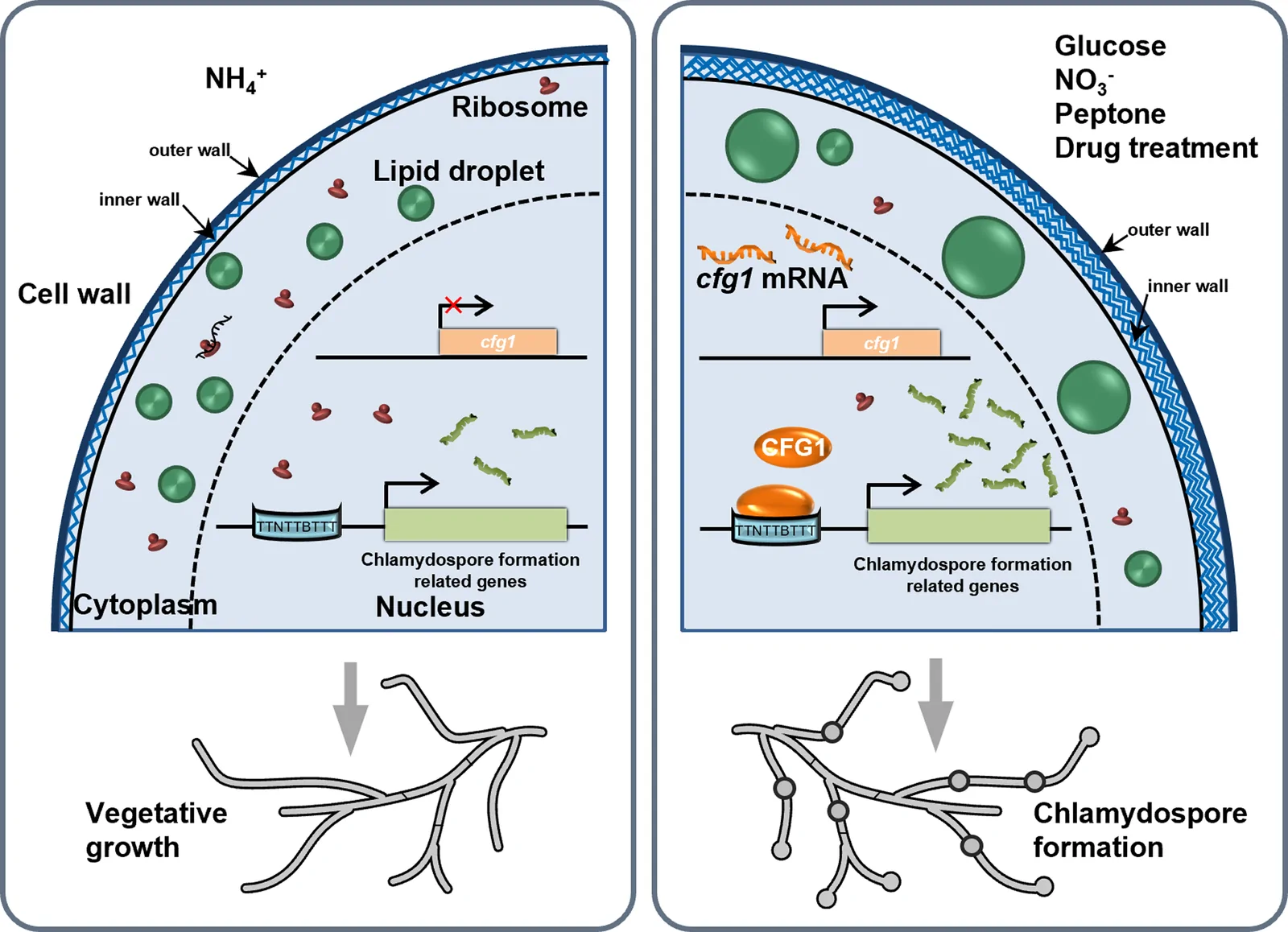
Model for CFG1 function in the control of chlamydospore formation in *Trichoderma*. Under specific environmental stimuli, such as glucose, nitrate, peptone, or certain drug treatments, the expression level of the transcriptional regulator CFG1 increases, whereas in an NH_4_^+^ environment, CFG1 expression does not increase, and only vegetative growth is maintained. When CFG1 expression is upregulated, it directly promotes the expression of chlamydospore formation-related genes and indirectly enhances the expression of lipid metabolism-related genes. This regulatory cascade promotes lipid droplet accumulation in the cytoplasm and drives the thickening of the inner cell wall. Simultaneously, ribosome expression decreases, causing the cell to enter a quiescent state. These coordinated changes ultimately drive the morphological transition from vegetative growth to chlamydospore formation.

Collectively, under chlamydospore-inducing conditions (e.g., peptone or nitrate as nitrogen sources), CFG1 expression is progressively upregulated and the CFG1 protein translocates to the nucleus, where it binds to specific DNA motifs in the promoter regions of target genes (Fig. 6). CFG1 directly regulates genes involved in diverse biological processes. In parallel, CFG1 indirectly influences glycerolipid metabolism, promoting the conversion of DAG to TAG and facilitating lipid droplet accumulation. These CFG1-mediated regulatory events collectively drive the morphological and physiological reprogramming required for chlamydospore formation, including cell wall remodeling (chitin deposition and double-layer formation), lipid droplet accumulation, nuclear consolidation, and ultimately entry into dormancy.

## MATERIALS AND METHODS

### Strains, medium, and growth conditions

The wild type and all constructed strains were inoculated on potato dextrose agar (PDA) medium (90 mm plate) and cultured under light for 120 h. Conidia were collected in distilled water and used as a spore solution. The spore solution was inoculated into a liquid medium with a final concentration of 10^5^ spores/mL. All strains were incubated at 28°C on a rotary shaker (170 rpm) in the dark. The liquid medium was based on minimal medium (MM) (1% [wt/vol] glucose, 6% [wt/vol] NaNO_3_), replacing the carbon and nitrogen sources or adding antifungal agents. The carbon sources included sucrose, galactose, and maltose, while the nitrogen sources included ammonium sulfate, peptone, and urea.

### Strain construction

The Δ*cfg1*, Δ*cfg2*, ΔOPB45020, Δ*cfg1-N*, Δ*cfg1-C,* Δ*dgat*, and Δ*pdat* deletion mutants were constructed as described previously (66). In brief, the hygromycin phosphotransferase gene (*hph*) cassette and the upstream and downstream fragments of the target gene were amplified via PCR and cloned into the vector pUC19 using the ClonExpress MultiS One Step Cloning Kit (Vazyme, China), yielding a plasmid for gene knockout. The deletion cassettes were amplified from the plasmids and used for transformation. Positive transformants were confirmed by PCR. The genes encoding CFG1 and eGFP (green fluorescent protein) were amplified separately via PCR, and a synthetic linker (3 × GGGGS) was amplified and used to conjugate *cfg1* and *gfp* at the C-terminus of *cfg1*. The *cfg1::egfp* cassette was then amplified and transformed into WT. The positive transformants were confirmed by PCR. The CFG1 complementation construct, geneticin gene (G-418) cassette, targeted gene, and downstream fragments of the targeted gene were amplified via PCR and cloned into the vector pUC19. The CFG1 overexpression construct was used to conjugate pcDNA and *cfg1* at the N-terminus of *cfg1*. The positive transformants were confirmed by PCR.

### Quantification of chlamydospore

The WT and all mutants were inoculated in a liquid medium for 120 h. A 1 mL mixture of hyphae and medium was harvested and resuspended in 9 mL of distilled water. Chlamydospores were prepared by breaking them using an ultrasonic crusher. Sonication was performed at 30% amplitude for short intervals (5 s on/5 s off) for 2–4 min. Three biological and technical replicates were performed for each culture, and the assay was repeated thrice.

### Chlamydospore staining and microscopy

The WT strain was grown in peptone medium at 170 rpm and 28°C in the dark with agitation for 12, 24, 36, 48, 60, 72, 84, and 96 h. The mycelium was rinsed with 1 × PBS and incubated in the dark either with 2 μg/mL BODIPY 493/505 (Invitrogen) for 30 min or 10 μg/mL Hoechst (Beyotime) for 20 min. The mycelium was rinsed twice with 1 × PBS (pH 7.4). The samples were observed using a confocal laser scanning microscope (Leica TCS SP8, Germany) equipped with 20×, 40×, 63×, and 100× lenses. The excitation wavelength of the BODIPY signals was set at 503 nm, and the emission was set in the wavelength range of 500–520 nm. Hoechst signal excitation wavelength was set at 352 nm, and emission was set at a wavelength range of 450–470 nm. The excitation wavelength of the GFP signal was set at 488 nm, and the emission was set at a wavelength range of 500–550 nm. Image processing was performed using Adobe Photoshop 2022. The mycelium was washed twice with ice-cold PBS and fixed with 2.5% glutaraldehyde in 0.1 M sodium cacodylate buffer (pH 7.4) for 2 h at 4°C. After fixation, the samples were washed three times with sodium cacodylate buffer and post-fixed with 1% osmium tetroxide for 1 h at room temperature. Subsequently, the samples were dehydrated using a graded series of ethanol solutions (30%, 50%, 70%, 90%, and 100%) and infiltrated with a 1:1 mixture of epoxy resin and propylene oxide for 1 h, followed by pure epoxy resin for 2 h. The samples were then embedded in epoxy resin molds and polymerized at 60°C for 48 h. Ultrathin sections (60–70 nm) were cut using a diamond knife on an ultramicrotome and collected on copper grids. The sections were stained with uranyl acetate and lead citrate for contrast. The samples were observed using TEM (Hitachi HT7800) operating at an acceleration voltage of 80 kV. TEM images were captured using a CCD camera.

### Determination of chitin content

The WT strain was grown in peptone medium at 28°C in the dark with agitation (170 rpm) for 1, 2, 3, and 4 days. The mycelium was freeze-dried using a lyophilizer (Powerdry LL3000) and ground in liquid nitrogen. For hydrolysis, the samples were subjected to 1 mL of 6 M HCl at 100°C for 4 h. After cooling, 0.2 mL of the hydrolysate was mixed with 0.25 mL 4% (vol/vol) acetylacetone solution (acetylacetone dissolved in 1.25 M sodium carbonate) and heated at 90°C for 1 h. To dissolve the precipitate, 2 mL of ethanol was added, and the solution was supplemented with 0.25 mL of Ehrlich reagent (1.6 g of N, N-dimethyl-p-aminobenzaldehyde dissolved in 60 mL of a 1:1 mixture of ethanol and concentrated HCl). The absorbance of the samples at 530 nm was measured to determine the chitin content. A standard curve was generated using commercial d-glucosamine. Three biological and technical replicates were used for treatment, and the assay was repeated thrice.

### RNA isolation and real-time PCR

Strains were grown in MM medium at 28°C with shaking at 170 rpm in the dark for 12, 36, 24, 48, and 72 h. The mycelium was harvested under dim-green light and immediately frozen in liquid nitrogen. The mycelium was ground to a fine powder using a mortar and pestle in liquid nitrogen, ensuring that the tissue did not thaw during grinding. Total RNA was extracted from each sample using the SteadyPure Plant RNA Extraction Kit (Accurate Biotechnology, China), and ~1 µg of total RNA was used for cDNA synthesis using Hifair III 1st Strand cDNA Synthesis SuperMix for qPCR (gDNA digester plus) (Yeasen). qPCR was performed using Hieff UNICON Universal Blue qPCR SYBR Green Master Mix (Yeasen), and the expression level of each gene was normalized to the translation elongation factor 1 alpha (*tef1*) gene. Statistical analyses were performed based on three biological and technical replicates, and the assays were repeated three times.

### RNA sequencing and analysis

Chlamydospore formation was observed using a microscope every 1–2 h, over the course of 5 days. Four sampling time points in MM medium were selected, and the samples were collected with three biological replicates at 48, 72, 96, and 120 h. Four sampling time points in peptone medium were selected, and the samples were collected with three biological replicates at 24, 48, 72, and 96 h. The samples were labeled S1 (Stage 1, mycelial growth phase), S2 (Stage 2, early chlamydospore formation), S3 (Stage 3, intercalary chlamydospore formation with lipid droplets beginning to aggregate), and S4 (Stage 4, chlamydospore maturation phase with complete cell wall thickening and fully aggregated lipid droplets). The mycelium was harvested under dim-green light and immediately frozen in liquid nitrogen. Transcriptome sequencing was performed using NovaSeq by Gene Denovo Biotechnology (Guangzhou, China). Three biological replicates from each group were used for transcriptome sequencing. After quality control, clean reads were mapped to the reference genome of *T. guizhouense* using HASAT2 v2.1.0. For each transcript, a fragment per kilobase of transcript per million mapped reads value was calculated to quantify its expression abundance and variations using StringTie v1.3.1. DEGs were identified using DEGseq2 with thresholds of |log_2_ (fold change)| ≥ 1 and FDR (https://www.genome.jp/kegg/). Venn diagrams and heatmaps were generated using the R environment.

### DAP-seq analysis

The cDNA sequence encoding the protein CFG1 was constructed in the Halo Tag plasmid. The open reading frame of *cfg1* was amplified from cDNA of the WT strain. This was inserted into the DB3 vector with a Halo-tag, the construct vector pCFG1-halo was identified via sequencing, and the vector and genomic DNA of *T. guizhouense* were sent to Genedenovo Biotechnology (Guangzhou, China) for DAP-Seq analysis. The CFG1 protein binds to the target genome fragment, and the DNA fragment of the genome bound by the target protein can be enriched, with the input group used as the control. In combination with high-throughput sequencing technology, DNA products after DAP were sequenced and analyzed, DNA binding sites of target proteins were searched from the whole genome, and high-throughput data were obtained using efficient sequencing methods. The DNA libraries were sequenced on an Illumina sequencing platform by Genedenovo Biotechnology Co. MEME-ChIP (https://meme-suite.org/meme/doc/meme-chip-output-format.html) was used to detect significant motif sequences in peak sequences.

### Metabolomic comparison analysis

WT and mutant strains in peptone medium were selected, and the samples were collected with four biological replicates at 48 h. The mycelium was harvested under dim-green light and immediately frozen in liquid nitrogen (to quench lipid metabolites). Based on a targeted metabolomics platform, the lipids were extracted and analyzed using ultra-high-performance liquid chromatography-quadrupole time-of-flight mass spectrometry (UHPLC-QTOF-MS), which was conducted by Shanghai OE Biotech Co., Ltd. (Shanghai, China); details are provided in the Supporting Information.

### Triglyceride assay

The mycelium was collected using the method described above, and the excess liquid was removed after rinsing with distilled water. The mixture was then homogenized using a mortar and pestle. The triglyceride content of the debris was measured using a commercial triglyceride assay kit (Applygen E1013-50) according to the manufacturer’s instructions. Each strain was assayed in triplicate. Statistical analyses were performed using three biological and technical replicates.

### Statistical analysis

Data are presented as mean ± standard deviation (SD) from three biological and technical replicates. Each experiment was performed three times. Statistical analysis was performed using one-way ANOVA followed by Tukey’s test in GraphPad Prism 8. In all bar graphs, statistical significance between groups is indicated by lowercase letters: groups that do not share any common letter are significantly different from each other at the *P* < 0.05 level. Venn diagrams and heatmaps were generated in the R environment.

## Data Availability

All data needed to evaluate the conclusions in the paper are present in the paper and the supplemental material. The transcriptome sequencing data are available in the NCBI Sequence Read Archive (SRA) under accession number PRJNA1454912.

## SUPPLEMENTAL MATERIAL

Figure S1 aem.00867-26-s0001.tif Dynamic changes in GO terms in DEGs during chlamydospore formation.

Figure S2 aem.00867-26-s0002.tif Comparative KEGG enrichment analysis of DEGs during chlamydospore formation.

Figure S3 aem.00867-26-s0003.tif Quantification of six transcription factors in different nitrogen sources.

Figure S4 aem.00867-26-s0004.tif Functional verification of *cfg1* and *cfg2* genes.

Figure S5 aem.00867-26-s0005.tif Identification of a conserved binding motif for CFG1.

Supplemental legends aem.00867-26-s0006.docx Legends for Fig. S1 to S5.

ASM does not own the copyrights to Supplemental Material that may be linked to, or accessed through, an article. The authors have granted ASM a non-exclusive, world-wide license to publish the Supplemental Material files. Please contact the corresponding author directly for reuse.

## References

[1] Wyatt TT, Wösten HAB, Dijksterhuis J. 2013. Fungal spores for dispersion in space and time. Adv Appl Microbiol 85:43–91. doi:10.1016/B978-0-12-407672-3.00002-2 23942148

[2] Deacon JW. 2013. Fungal spores, spore dormancy, and spore dispersal, p 184–212. *In* Fungal biology. John Wiley & Sons.

[3] Lin X, Heitman J. 2005. Chlamydospore formation during hyphal growth in *Cryptococcus neoformans*. Eukaryot Cell 4:1746–1754. doi:10.1128/EC.4.10.1746-1754.2005 PMC1265899 16215181

[4] Francisco CS, Ma X, Zwyssig MM, McDonald BA, Palma-Guerrero J. 2019. Morphological changes in response to environmental stresses in the fungal plant pathogen *Zymoseptoria tritici*. Sci Rep 9:9642. doi:10.1038/s41598-019-45994-3 PMC6610121 31270361

[5] Couteaudier Y, Alabouvette C. 1990. Survival and inoculum potential of conidia and chlamydospores of *Fusarium oxysporum* f.sp. lini in soil. Can J Microbiol 36:551–556. doi:10.1139/m90-096 2245379

[6] Stevenson IL, Becker SAW. 1972. The fine structure and development of chlamydospores of *Fusarium oxysporum*. Can J Microbiol 18:997–1002. doi:10.1139/m72-155 4560921

[7] Ohara T, Tsuge T. 2004. *FoSTUA*, encoding a basic helix-loop-helix protein, differentially regulates development of three kinds of asexual spores, macroconidia, microconidia, and chlamydospores, in the fungal plant pathogen *Fusarium oxysporum*. Eukaryot Cell 3:1412–1422. doi:10.1128/EC.3.6.1412-1422.2004 PMC539018 15590816

[8] Bai G, Shaner G. 2004. Management and resistance in wheat and barley to fusarium head blight. Annu Rev Phytopathol 42:135–161. doi:10.1146/annurev.phyto.42.040803.140340 15283663

[9] Martin SW, Douglas LM, Konopka JB. 2005. Cell cycle dynamics and quorum sensing in *Candida albicans* chlamydospores are distinct from budding and hyphal growth. Eukaryot Cell 4:1191–1202. doi:10.1128/EC.4.7.1191-1202.2005 PMC1168967 16002645

[10] Jansons VK, Nickerson WJ. 1970. Induction, morphogenesis, and germination of the chlamydospore of *Candida albicans*. J Bacteriol 104:910–921. doi:10.1128/jb.104.2.910-921.1970 PMC285075 4099098

[11] Cole GT, Seshan KR, Phaneuf M, Lynn KT. 1991. Chlamydospore-like cells of *Candida albicans* in the gastrointestinal tract of infected, immunocompromised mice. Can J Microbiol 37:637–646. doi:10.1139/m91-108 1954577

[12] Navarathna DHMLP, Pathirana RU, Lionakis MS, Nickerson KW, Roberts DD. 2016. *Candida albicans* ISW2 regulates chlamydospore suspensor cell formation and virulence *in vivo* in a mouse model of disseminated candidiasis. PLoS One 11:e0164449. doi:10.1371/journal.pone.0164449 PMC5058487 27727302

[13] Blair J, Biddle A. 2020. Stimulating *Duddingtonia flagrans* chlamydospore production through dehydration. Parasitol Res 119:123–128. doi:10.1007/s00436-019-06499-0 PMC6942027 31735994

[14] Spraker JE, Sanchez LM, Lowe TM, Dorrestein PC, Keller NP. 2016. *Ralstonia solanacearum* lipopeptide induces chlamydospore development in fungi and facilitates bacterial entry into fungal tissues. ISME J 10:2317–2330. doi:10.1038/ismej.2016.32 PMC4989320 26943626

[15] Nguyen VQ, Sil A. 2008. Temperature-induced switch to the pathogenic yeast form of *Histoplasma capsulatum* requires Ryp1, a conserved transcriptional regulator. Proc Natl Acad Sci USA 105:4880–4885. doi:10.1073/pnas.0710448105 PMC2290814 18339808

[16] Zordan RE, Galgoczy DJ, Johnson AD. 2006. Epigenetic properties of white-opaque switching in *Candida albicans* are based on a self-sustaining transcriptional feedback loop. Proc Natl Acad Sci USA 103:12807–12812. doi:10.1073/pnas.0605138103 PMC1535343 16899543

[17] Lohse MB, Zordan RE, Cain CW, Johnson AD. 2010. Distinct class of DNA-binding domains is exemplified by a master regulator of phenotypic switching in *Candida albicans*. Proc Natl Acad Sci USA 107:14105–14110. doi:10.1073/pnas.1005911107 PMC2922561 20660774

[18] Huang G, Wang H, Chou S, Nie X, Chen J, Liu H. 2006. Bistable expression of WOR1, a master regulator of white-opaque switching in *Candida albicans*. Proc Natl Acad Sci USA 103:12813–12818. doi:10.1073/pnas.0605270103 PMC1540355 16905649

[19] Srikantha T, Borneman AR, Daniels KJ, Pujol C, Wu W, Seringhaus MR, Gerstein M, Yi S, Snyder M, Soll DR. 2006. TOS9 regulates white-opaque switching in *Candida albicans*. Eukaryot Cell 5:1674–1687. doi:10.1128/EC.00252-06 PMC1595353 16950924

[20] Hernday AD, Lohse MB, Fordyce PM, Nobile CJ, DeRisi JL, Johnson AD. 2013. Structure of the transcriptional network controlling white-opaque switching in *Candida albicans*. Mol Microbiol 90:22–35. doi:10.1111/mmi.12329 PMC3888361 23855748

[21] Pande K, Chen C, Noble SM. 2013. Passage through the mammalian gut triggers a phenotypic switch that promotes *Candida albicans* commensalism. Nat Genet 45:1088–1091. doi:10.1038/ng.2710 PMC3758371 23892606

[22] Michielse CB, van Wijk R, Reijnen L, Manders EMM, Boas S, Olivain C, Alabouvette C, Rep M. 2009. The nuclear protein Sge1 of *Fusarium oxysporum* is required for parasitic growth. PLoS Pathog 5:e1000637. doi:10.1371/journal.ppat.1000637 PMC2762075 19851506

[23] Chen Y, Zhai S, Zhang H, Zuo R, Wang J, Guo M, Zheng X, Wang P, Zhang Z. 2014. Shared and distinct functions of two Gti1/Pac2 family proteins in growth, morphogenesis and pathogenicity of *Magnaporthe oryzae*. Environ Microbiol 16:788–801. doi:10.1111/1462-2920.12204 23895552

[24] Tollot M, Assmann D, Becker C, Altmüller J, Dutheil JY, Wegner C-E, Kahmann R. 2016. The WOPR protein Ros1 is a master regulator of sporogenesis and late effector gene expression in the maize pathogen *Ustilago maydis*. PLoS Pathog 12:e1005697. doi:10.1371/journal.ppat.1005697 PMC4917244 27332891

[25] Contreras-Cornejo HA, Macías-Rodríguez L, Cortés-Penagos C, López-Bucio J. 2009. Trichoderma virens, a plant beneficial fungus, enhances biomass production and promotes lateral root growth through an auxin-dependent mechanism in Arabidopsis. Plant Physiol 149:1579–1592. doi:10.1104/pp.108.130369 PMC2649400 19176721

[26] Lorito M, Woo SL, Harman GE, Monte E. 2010. Translational research on *Trichoderma*: from ‘Omics to the field. Ann Rev Phytopathol 48:395–417. doi:10.1146/annurev-phyto-073009-114314 20455700

[27] Tripathi P, Singh PC, Mishra A, Chauhan PS, Dwivedi S, Bais RT, Tripathi RD. 2013. *Trichoderma*: a potential bioremediator for environmental clean up. Clean Techn Environ Policy 15:541–550. doi:10.1007/s10098-012-0553-7

[28] Zhu X, Wang Y, Wang X, Wang W. 2022. Exogenous regulators enhance the yield and stress resistance of chlamydospores of the biocontrol agent *Trichoderma harzianum* T4. J Fungi 8:1017. doi:10.3390/jof8101017 PMC9604748 36294583

[29] Hernández-Cervantes A, Znaidi S, van Wijlick L, Denega I, Basso V, Ropars J, Sertour N, Sullivan D, Moran G, Basmaciyan L, Bon F, Dalle F, Bougnoux M-E, Boekhout T, Yang Y, Li Z, Bachellier-Bassi S, d’Enfert C. 2020. A conserved regulator controls asexual sporulation in the fungal pathogen *Candida albicans*. Nat Commun 11:6224. doi:10.1038/s41467-020-20010-9 PMC7718266 33277479

[30] Wang B-B, Wang F-H, Wang Y-J, Jia Y-B, Tian S-Y, Zhang X-C, Xue Y-J, Li Y-L, Cai K-Z. 2024. Microstructure characterization of different types of chlamydospores in *Arthrobotrys flagrans*. J Basic Microbiol 64:32–41. doi:10.1002/jobm.202300308 37699751

[31] Cuadrado Osorio PD, Castillo Saldarriaga CR, Cubide Cardenas JA, Gómez Alvarez MI, Bautista Bautista EJ. 2021. Chlamydospores production enhancement of *Duddingtonia flagrans* in a solid-state fermentation system. DYNA 88:152–159. doi:10.15446/dyna.v88n216.85978 32638326

[32] Venkatesh N, Greco C, Drott MT, Koss MJ, Ludwikoski I, Keller NM, Keller NP. 2022. Bacterial hitchhikers derive benefits from fungal housing. Curr Biol 32:1523–1533. doi:10.1016/j.cub.2022.02.017 PMC9009100 35235767

[33] Spraker JE, Wiemann P, Baccile JA, Venkatesh N, Schumacher J, Schroeder FC, Sanchez LM, Keller NP. 2018. Conserved responses in a war of small molecules between a plant-pathogenic bacterium and fungi. mBio 9:e00820-18. doi:10.1128/mBio.00820-18 PMC5964348 29789359

[34] Goh YK, Daida P, Vujanovic V. 2009. Effects of abiotic factors and biocontrol agents on chlamydospore formation in *Fusarium graminearum* and *Fusarium sporotrichioides*. Biocontrol Sci Technol 19:151–167. doi:10.1080/09583150802627033

[35] Gemin O, Gluc M, Rosa H, Purdy M, Niemann M, Peskova Y, Mattei S, Jomaa A. 2024. Ribosomes hibernate on mitochondria during cellular stress. Nat Commun 15:8666. doi:10.1038/s41467-024-52911-4 PMC11461667 39379376

[36] Koli S, Shetty S. 2024. Ribosomal dormancy at the nexus of ribosome homeostasis and protein synthesis. BioEssays 46:e2300247. doi:10.1002/bies.202300247 38769702

[37] Lohse MB, Rosenberg OS, Cox JS, Stroud RM, Finer-Moore JS, Johnson AD. 2014. Structure of a new DNA-binding domain which regulates pathogenesis in a wide variety of fungi. Proc Natl Acad Sci USA 111:10404–10410. doi:10.1073/pnas.1410110111 PMC4115540 24994900

[38] Zhang S, Zhang T, Yan M, Ding J, Chen J. 2014. Crystal structure of the WOPR-DNA complex and implications for Wor1 function in white-opaque switching of *Candida albicans*. Cell Res 24:1108–1120. doi:10.1038/cr.2014.102 PMC4152732 25091450

[39] Yan M, Nie X, Wang H, Gao N, Liu H, Chen J. 2015. SUMOylation of Wor1 by a novel SUMO E3 ligase controls cell fate in *Candida albicans*. Mol Microbiol 98:69–89. doi:10.1111/mmi.13108 26112173

[40] John E, Singh KB, Oliver RP, Tan K-C. 2021. Transcription factor control of virulence in phytopathogenic fungi. Mol Plant Pathol 22:858–881. doi:10.1111/mpp.13056 PMC8232033 33973705

[41] Bartlett A, O’Malley RC, Huang S-SC, Galli M, Nery JR, Gallavotti A, Ecker JR. 2017. Mapping genome-wide transcription-factor binding sites using DAP-seq. Nat Protoc 12:1659–1672. doi:10.1038/nprot.2017.055 PMC5576341 28726847

[42] Dahlqvist A, Stahl U, Lenman M, Banas A, Lee M, Sandager L, Ronne H, Stymne S. 2000. Phospholipid:diacylglycerol acyltransferase: an enzyme that catalyzes the acyl-CoA-independent formation of triacylglycerol in yeast and plants. Proc Natl Acad Sci USA 97:6487–6492. doi:10.1073/pnas.120067297 PMC18631 10829075

[43] Guan C, Niu Y, Chen S-C, Kang Y, Wu J-X, Nishi K, Chang CCY, Chang T-Y, Luo T, Chen L. 2020. Structural insights into the inhibition mechanism of human sterol *O*-acyltransferase 1 by a competitive inhibitor. Nat Commun 11:2478. doi:10.1038/s41467-020-16288-4 PMC7234994 32424158

[44] Löffler HJM, Schippers B. 1985. Inhibition of chlamydospore production in *Fusarium oxysporum* by ammonia and high ion concentrations. Can J Microbiol 31:508–512. doi:10.1139/m85-095

[45] Twumasi-Boateng K, Yu Y, Chen D, Gravelat FN, Nierman WC, Sheppard DC. 2009. Transcriptional profiling identifies a role for BrlA in the response to nitrogen depletion and for StuA in the regulation of secondary metabolite clusters in *Aspergillus fumigatus*. Eukaryot Cell 8:104–115. doi:10.1128/EC.00265-08 PMC2620752 19028996

[46] Oh Y, Donofrio N, Pan H, Coughlan S, Brown DE, Meng S, Mitchell T, Dean RA. 2008. Transcriptome analysis reveals new insight into appressorium formation and function in the rice blast fungus *Magnaporthe oryzae*. Genome Biol 9:R85. doi:10.1186/gb-2008-9-5-r85 PMC2441471 18492280

[47] Gow NAR, Latge J-P, Munro CA. 2017. The fungal cell wall: structure, biosynthesis, and function. Microbiol Spectr 5:10. doi:10.1128/microbiolspec.funk-0035-2016 PMC11687499 28513415

[48] Román E, Prieto D, Hidalgo-Vico S, Alonso-Monge R, Pla J. 2023. The defective gut colonization of *Candida albicans hog1* MAPK mutants is restored by overexpressing the transcriptional regulator of the white opaque transition *WOR1*. Virulence 14:2174294. doi:10.1080/21505594.2023.2174294 PMC9928469 36760104

[49] Dai B, Xu Y, Gao N, Chen J. 2021. Wor1-regulated ferroxidases contribute to pigment formation in opaque cells of *Candida albicans*. FEBS Open Bio 11:598–621. doi:10.1002/2211-5463.13070 PMC7931227 33350590

[50] Hidalgo-Vico S, Casas J, García C, Lillo MP, Alonso-Monge R, Román E, Pla J. 2022. Overexpression of the white opaque switching master regulator Wor1 alters lipid metabolism and mitochondrial function in *Candida albicans*. J Fungi (Basel) 8:1028. doi:10.3390/jof8101028 PMC9604646 36294593

[51] Conway TP, Conway K, Boksa FA, Pujol C, Wessels D, Soll DR. 2021. Role of the *WOR1* promoter of *Candida albicans* in opaque commitment. mBio 12:e0232021. doi:10.1128/mBio.02320-21 PMC8546583 34488444

[52] Luo Z, Xiong D, Tian C. 2024. The roles of Gti1/Pac2 family proteins in fungal growth, morphogenesis, stress response, and pathogenicity. Mol Plant Microbe Interact 37:488–497. doi:10.1094/MPMI-11-23-0198-CR 38427716

[53] Hu X, Li B, Li Y, Xia Y, Jin K. 2025. MaPac2, a transcriptional regulator, is involved in conidiation, stress tolerances and pathogenicity in *Metarhizium acridum*. J Fungi 11:100. doi:10.3390/jof11020100 PMC11855946 39997395

[54] Paes HC, Derengowski L da S, Peconick LDF, Albuquerque P, Pappas GJ, Nicola AM, Silva FBA, Vallim MA, Alspaugh JA, Felipe MSS, Fernandes L. 2018. A Wor1-like transcription factor is essential for virulence of *Cryptococcus neoformans*. Front Cell Infect Microbiol 8:369. doi:10.3389/fcimb.2018.00369 PMC6243373 30483479

[55] Alkafeef SS, Yu C, Huang L, Liu H. 2018. Wor1 establishes opaque cell fate through inhibition of the general co-repressor Tup1 in *Candida albicans*. PLoS Genet 14:e1007176. doi:10.1371/journal.pgen.1007176 PMC5786334 29337983

[56] Guan Z, Liu H. 2015. The WOR1 5’ untranslated region regulates white-opaque switching in *Candida albicans* by reducing translational efficiency. Mol Microbiol 97:125–138. doi:10.1111/mmi.13014 PMC4552353 25831958

[57] Zordan RE, Miller MG, Galgoczy DJ, Tuch BB, Johnson AD. 2007. Interlocking transcriptional feedback loops control white-opaque switching in *Candida albicans*. PLoS Biol 5:e256. doi:10.1371/journal.pbio.0050256 PMC1976629 17880264

[58] Okmen B, Collemare J, Griffiths S, van der Burgt A, Cox R, de Wit PJGM. 2014. Functional analysis of the conserved transcriptional regulator CfWor1 in *Cladosporium fulvum* reveals diverse roles in the virulence of plant pathogenic fungi. Mol Microbiol 92:10–27. doi:10.1111/mmi.12535 24521437

[59] Jonkers W, Dong Y, Broz K, Kistler HC. 2012. The Wor1-like protein Fgp1 regulates pathogenicity, toxin synthesis and reproduction in the phytopathogenic fungus *Fusarium graminearum*. PLoS Pathog 8:e1002724. doi:10.1371/journal.ppat.1002724 PMC3364952 22693448

[60] Vela-Corcía D, Aditya Srivastava D, Dafa-Berger A, Rotem N, Barda O, Levy M. 2019. MFS transporter from *Botrytis cinerea* provides tolerance to glucosinolate-breakdown products and is required for pathogenicity. Nat Commun 10:2886. doi:10.1038/s41467-019-10860-3 PMC6599007 31253809

[61] Calmes B, Morel-Rouhier M, Bataillé-Simoneau N, Gelhaye E, Guillemette T, Simoneau P. 2015. Characterization of glutathione transferases involved in the pathogenicity of *Alternaria brassicicola*. BMC Microbiol 15:123. doi:10.1186/s12866-015-0462-0 PMC4470081 26081847

[62] He R, Liu J, Zhang S, Wang M, Zhang Y, Liang X, Yang Y. 2026. Molecular mechanism of glutathione S-transferase involved in resistance to multiple fungicide in *Lasiodiplodia theobromae*. Pestic Biochem Physiol 216:106759. doi:10.1016/j.pestbp.2025.106759 41350054

[63] Feng Z, Liu X, Zhu H, Yao Q. 2020. Responses of arbuscular mycorrhizal symbiosis to abiotic stress: a lipid-centric perspective. Front Plant Sci 11:578919. doi:10.3389/fpls.2020.578919 PMC7688922 33281845

[64] Olsson PA, Johansen A. 2000. Lipid and fatty acid composition of hyphae and spores of arbuscular mycorrhizal fungi at different growth stages. Mycol Res 104:429–434. doi:10.1017/S0953756299001410

[65] Li J, Cao J, Chen H, Tang X, Zhang H, Chen W. 2022. Functional characterization of two diacylglycerol acyltransferase 1 genes in *Mortierella alpina*. Lett Appl Microbiol 74:194–203. doi:10.1111/lam.13597 34755357

[66] Zhang J, Miao Y, Rahimi MJ, Zhu H, Steindorff A, Schiessler S, Cai F, Pang G, Chenthamara K, Xu Y, Kubicek CP, Shen Q, Druzhinina IS. 2019. Guttation capsules containing hydrogen peroxide: an evolutionarily conserved NADPH oxidase gains a role in wars between related fungi. Environ Microbiol 21:2644–2658. doi:10.1111/1462-2920.14575 PMC6850483 30815928

