# Supplementary material for: The Gti1/Pac2 family protein CFG1 controls fungal chlamydospore formation through orchestrating cell wall remodeling, lipid metabolism, and ribosome biogenesis
Source: Appl Environ Microbiol. 2026 Aug 5;92(9):e00867-26. doi: 10.1128/aem.00867-26 (PMC13599505; doi:10.1128/aem.00867-26)
Supplement: Supplementary file 1 [file aem.00867-26-s0006.docx]

Fig. S1. Dynamic changes in GO terms in DEGs during chlamydospore formation.

Gene Ontology (GO) enrichment analysis of DEGs in NO₃⁻ medium. Each diagram represents the top 20 pathways based on enrichment significance. Pairwise comparisons were made the S1, S2, S3, and S4 stages. **A**, S2 vs S1; **B**, S3 vs S2; **C**, S4 vs S3.

Fig. S2. Comparative KEGG enrichment analysis of DEGs during chlamydospore formation.

Top 30 significantly enriched Kyoto Encyclopedia of Genes and Genomes (KEGG) pathways of DEGs in S2, S3, and S4. **A**, NO₃⁻ medium. **B**, peptone medium. Top 20 significantly enriched KEGG pathways of DEGs in S3 and S4 in the NO₃⁻ medium. **C**, up-regulated DEGs. **D**, downregulated DEGs.

Fig. S3. Quantification of 6 transcription factors in different nitrogen sources.

Expression levels of OPB38432, OPB37199, OPB39396, OPB36447, OPB45677, and OPB43668 in NO₃⁻ **A**, peptone **B**, and ammonium sulfate **C** media after 3 days. Error bars indicate the SD of three biological replicates. Statistical analysis was performed using one-way ANOVA followed by Tukey’s test, and different lowercase letters represent significant differences (*P* < 0.05). Groups not sharing any common letter are significantly different from each other.

Fig. S4. Functional verification of *cfg1* and *cfg2* genes.

Amplification was performed using gene-specific primers to detect target sequences in different samples. **A**, Lanes: M, DNA molecular weight marker, 1-4, experimental samples; 5, *cfg1* gene; 6, *cfg2* gene. **B**, Lanes: M, DNA molecular weight marker, 1-4, experimental samples; 5, *cfg1-N* gene; 6, *cfg1-C* gene. **C**, Phenotype of Δ*cfg1*-mutant on nitrogen source medium supplemented with MF and AF. Phenotypes of Δ*cfg1^OE^* and Δ*cfg1^C^* strains in nitrogen source medium. Scale bar, 15 μm. **D**, Phenotype of WT and Δ*cfg2*-mutant in nitrogen source medium at 48 h. Scale bar, 15 μm. **E**, Fluorescence intensity profile (source: white arrow in [Fig. 4E]) of CFG1eGFP. The numbers represent the quantification of green signals using ImageJ.

Fig. S5. Identification of a conserved binding motif for CFG1.

**A**, Conserved motifs identiﬁed by CFG1 DAP-seq. **B**, ChIP-qPCR analysis showing the lack of enrichment at the predicted binding motif. Error bars indicate the SD of three biological replicates. Significant differences were evaluated using Student’s *t*-test: **P* < 0.05; ns, not significant.
